# Can ChatGPT Boost Students’ Employment Confidence? A Pioneering Booster for Career Readiness

**DOI:** 10.3390/bs15030362

**Published:** 2025-03-14

**Authors:** Yu Xiao, Li Zheng

**Affiliations:** 1 Institute of Education, Tsinghua University, Beijing 100084, China; xiaoyu921@tsinghua.edu.cn; 2 School of Education, Shanghai Normal University, Shanghai 200234, China

**Keywords:** ChatGPT, university student, employment confidence, inverse probability weighting, structural equation modeling

## Abstract

This study examines the impact of ChatGPT on university students’ employment confidence, utilizing comprehensive methodologies such as regression analysis, Inverse Probability Weighting (IPW), and Structural Equation Modeling (SEM). The results indicate that the regular use of ChatGPT significantly enhances students’ confidence in securing employment, with stronger effects observed among undergraduate students and those in social sciences. Additionally, this study reveals that students’ experience with ChatGPT plays a partial mediating role in this effect, underscoring the importance of user interaction in realizing the benefits of AI tools. These findings suggest that ChatGPT not only improves cognitive abilities and career-related knowledge but also boosts students’ proactive job-seeking behaviors, fostering increased job market readiness. The implications are far-reaching, highlighting how AI tools can enhance career development support, particularly for students at earlier stages of their academic journey. As AI technologies continue to influence education, this study offers valuable insights into how such tools can effectively prepare students for the job market, potentially contributing to future research and shaping educational practices in ways that address employment challenges.

## 1. Introduction

The release of ChatGPT (https://chatgpt.com/) in November 2022 marked a significant milestone in the development of artificial intelligence (AI), catapulting AI technology into the global spotlight. With millions of users across the globe and extensive media coverage, AI-powered tools saw unprecedented growth, creating new opportunities in education, business, and beyond. ChatGPT, a language generation model developed by OpenAI, rapidly gained global attention for its natural language processing capabilities. This breakthrough in AI has sparked a revolution across various sectors (García-Alonso et al., 2024; Pérez-Núñez, 2023; Pack & Maloney, 2023). As AI technology continues to evolve, AI tools, especially generative models like ChatGPT, have infiltrated fields such as education and career development. These tools are revolutionizing academic and professional landscapes by enabling researchers, educators, and professionals to streamline workflows and enhance productivity, creativity, and access to valuable insights (Tang et al., 2024; Vinchon et al., 2024; J. Zhang et al., 2024).

Employment is a significant concern for college students, as many struggle to secure jobs after graduation. Recent studies highlight that the employment rate for recent college graduates in the U.S. has been declining, with only 40% of full-time undergraduate students employed in 2020 (National Center for Education Statistics, 2022). Additionally, the unemployment rate for recent graduates increased to 5.3% in the third quarter of 2024 (Federal Reserve Bank of New York, 2024). As a result, many students feel uncertain about their future career prospects; a survey revealed that nearly 66% of college students lack confidence in their ability to find a job after graduation (Inside Higher Ed, 2018). For college students, employment confidence directly affects students’ job-seeking attitudes, enthusiasm, and ultimate employment outcomes. Students with higher job confidence are more proactive in their job search and tend to secure employment more quickly (Aufa et al., 2024; Chen et al., 2023; Guan et al., 2013). Job search self-efficacy, a key component of employment confidence, has been shown to positively influence the number of job offers received (Liu et al., 2014; Moynihan et al., 2003). In addition, self-confidence is a predictor of job readiness, which is essential for effective job-seeking and achieving favorable employment outcomes (Ristiani & Lusianingrum, 2022; D. Wang et al., 2022).

ChatGPT has emerged as a versatile tool in higher education, offering significant benefits beyond academic support. Research indicates that ChatGPT can enhance students’ cognitive skills and career-relevant knowledge, with frequent usage positively impacting these areas, as high-quality ChatGPT outputs significantly improve cognitive skills and career-relevant knowledge (Essel et al., 2024; Suriano et al., 2025; Urban et al., 2024). In addition, ChatGPT provides personalized learning experiences that support cognitive development and career-relevant knowledge acquisition (Dahri et al., 2024). Beyond academics, ChatGPT assists students in career planning, job-seeking skills, and personal development, making it a comprehensive tool for student success (Bhullar et al., 2024). This multifaceted support helps students prepare for the job market and enhances their overall readiness for professional challenges.

Gender stereotypes, education systems, and economic conditions all shape employment confidence. Research shows that male candidates are often favored over equally qualified female candidates, particularly in STEM fields, where women report lower confidence despite high career commitment (Emeka, 2024; Ananthram et al., 2023). Education systems also play a crucial role—countries with vocational training models, like Germany and Switzerland, provide clear career pathways, whereas academic-driven systems, such as in the U.S., may leave students with fewer practical job skills (Sweet, 2012). Additionally, employment confidence varies by economic development; in developing countries, job market instability lowers confidence, while in developed economies, labor shortages create better opportunities for graduates (James, 2021). Given these challenges, AI tools, like ChatGPT, could help bridge gaps by providing career guidance, personalized job recommendations, and skill development support, making employment pathways more accessible and equitable.

Negative discussions surrounding AI have been found to significantly reduce students’ confidence in their expected earnings, making them feel more vulnerable to job displacement and its potential impact on career prospects (Huseynov, 2023; Thomson et al., 2024). While many students acknowledge ChatGPT’s capabilities, concerns persist regarding academic integrity violations and the changing job market, contributing to uncertainty about AI’s long-term influence on career opportunities (Al-Abdullatif & Alsubaie, 2024; García-López et al., 2025; Yu et al., 2024).

Additionally, overreliance on ChatGPT has been linked to declines in creativity and motivation, affecting students’ problem-solving abilities and independent thinking (Muñoz et al., 2023; Toma & Yánez-Pérez, 2024). This decline in creativity and motivation negatively impacts their academic performance and may result in a deficiency of critical job skills required for the future job market (Tanvir et al., 2023). These findings highlight the dual impact of AI—while it can enhance career confidence, excessive reliance may hinder skill acquisition—necessitating a balanced approach to AI integration in education.

While ChatGPT usage directly provides access to knowledge and assistance in task completion, the experience of using ChatGPT, including perceived ease, reliability, and satisfaction, may play a critical mediating role. This mediating effect is reflected in how users translate technological interaction into self-efficacy and optimism in their employability. Research suggests that user trust and experience with ChatGPT significantly influence behavioral outcomes, such as adoption and effectiveness (Choudhury & Shamszare, 2023). Moreover, satisfaction and interaction quality with ChatGPT have been identified as crucial factors shaping users’ attitudes toward its integration in diverse sectors (Sökmen et al., 2024).

Based on the current situation, understanding how AI tools, like ChatGPT, influence students’ confidence in securing employment provides valuable insights for educators, technology developers, and policymakers. This knowledge can help them create more effective strategies to address the challenges posed by the evolving job market, ensuring that students are better equipped with the skills and mindset needed to navigate future employment opportunities. By recognizing both the positive and negative impacts of AI, stakeholders can work together to optimize career preparation programs and foster a more resilient workforce.

## 2. Literature

### 2.1. The Role of AI Tools in Education

ChatGPT has truly transformed the landscape of personalized education by delivering real-time, adaptive learning experiences that are meticulously designed to meet the unique needs of each student. ChatGPT not only heightens student engagement but also significantly boosts academic performance (Deng et al., 2024; Heung & Chiu, 2025; Lo et al., 2024). By customizing educational content to align with individual learning requirements, ChatGPT paves the way for highly personalized educational journeys, targeting specific areas where students need improvement and ensuring that the learning process is more impactful and efficient (Akiba & Fraboni, 2023; Kabudi et al., 2021; S. Wang et al., 2024; Li, 2023; Sreen & Majid, 2024). Additionally, ChatGPT extends its educational support beyond mere academic content, offering comprehensive assistance in writing, tutoring, and providing personalized feedback that effectively addresses student doubts and cultivates a more profound comprehension of intricate subjects (Royani et al., 2024; Sok & Heng, 2024).

ChatGPT significantly extends its utility in the realm of career planning and guidance, offering tailored advice on job searches, career exploration, and professional development. ChatGPT not only aids in crafting resumes and preparing for interviews but also supports skill development, thereby becoming an invaluable asset for students mapping out their professional trajectories. By integrating ChatGPT into career advising, it democratizes access to expert guidance, thereby enhancing job readiness for students from varied backgrounds (Akiba & Fraboni, 2023; Crawford et al., 2023). Additionally, AI tools, like ChatGPT and Pathfinder, are employed in educational and job search settings, providing customized university and career guidance and optimizing the job search process with personalized recommendations and effective strategies (Deshmukh & Bajaj, 2024; Jain et al., 2024; Jawhar et al., 2024; Rajaram et al., 2024).

### 2.2. Students’ Employment Confidence

Employment confidence, a pivotal metric affecting career decisions and labor market entry, reflects individuals’ self-assessment of employability skills, job market knowledge, and their ability to meet employer expectations (Barron & Gravert, 2022; Qenani et al., 2014). High employment confidence correlates with better job prospects and psychological well-being during career transitions, spurring proactive career preparation and readiness for job opportunities, which, in turn, enhances the likelihood of career success (Creed et al., 2003; R. Zhang & Jen, 2024). This confidence is shaped by self-confidence, psychological well-being, pre-entry work experience, family support, and self-construal, with psychological well-being being a dominant predictor (Khairunnisa et al., 2022; Bennett et al., 2023; Inavatin et al., 2020).

Technology’s impact on employment confidence is multifaceted. Innovations in technology can bolster employment confidence by enhancing SMEs’ competitiveness and market adaptability, creating new opportunities and economic growth (Chege & Wang, 2020; Meier et al., 2025; S. Wang & Zhang, 2024), and by increasing productivity and creating new roles through automation (Aghion et al., 2021). However, technology also has negative implications, as rapid advancements in automation and robotics are linked to job insecurity and anxiety, particularly in sectors where technological change outpaces workers’ upskilling (Nam, 2019; Nikolova et al., 2024; Yam et al., 2023). Moreover, technological uncertainty and the potential for job displacement can lead to lower confidence levels, especially among workers with limited adaptability to new tools and systems (Brougham & Haar, 2020).

### 2.3. Differences in Technology Use Across Academic Levels and Fields

Studies reveal varying technology use among academic levels. Undergraduates commonly use devices like smartphones, laptops, and desktops mainly for social and general academic purposes but are less adept at using advanced digital tools for complex tasks (Cohen et al., 2022; Kwon et al., 2013; Sapci et al., 2021). Graduate students take a more strategic approach, prioritizing system and service quality for accessing high-quality resources, like digital libraries, and seek additional training (Xu & Du, 2019; Shaw & Giacquinta, 2000). Doctoral students focus on using technology for advanced research, including data management and virtual collaboration, though cultural mismatches can be problematic (Boulton, 2015). In essence, technology use patterns among students reflect their distinct academic needs and priorities at different stages (Rashid & Asghar, 2016).

Research reveals that students in social sciences, applied sciences, and arts and humanities exhibit different technology use patterns for academic purposes. Social science students frequently use technology, especially social media, for communication and socialization more than those in other fields (Campos et al., 2014). In contrast, applied science students rely heavily on technology for data collection, analysis, and solving technical problems, consistent with their practical and research-intensive curriculum (Moses et al., 2014). Arts and humanities students see technology as less critical but still important for accessing resources and creative work (Trusz, 2020). These varying patterns underscore how disciplinary norms and needs shape students’ technology engagement.

### 2.4. Ethical Considerations of AI

One of the most debated ethical challenges of AI is its potential to facilitate academic dishonesty. AI-powered tools, including ChatGPT, can generate essays, solve mathematical problems, and even assist in coding assignments, raising concerns about students bypassing traditional learning processes. While AI can be a valuable educational resource, its misuse has led to increasing calls for institutions to establish stricter policies on AI-assisted work. Kocak (2024) conducted a comprehensive review on publication ethics in the era of AI, focusing on the risks and challenges posed by AI-generated content in academic writing. The study identified key ethical issues such as bias, distortion, irrelevance, misrepresentation, and plagiarism in AI-generated texts. It emphasized that even when not used maliciously, AI outputs may unintentionally introduce inaccuracies due to flawed training data and lack of critical reasoning. Additionally, Koçak examined the need for AI disclosure policies, arguing that academic institutions must require authors to explicitly state if AI-assisted tools were used in their research and writing processes. The study also discussed retraction policies for AI-generated content, highlighting recent cases where AI-created misinformation led to article retractions. Based on these findings, Koçak proposed the development of new ethical standards and transparency guidelines to regulate AI’s role in scholarly work.

AI’s reliance on massive datasets for training raises serious legal and ethical questions regarding copyright infringement. AI models, including generative AI like ChatGPT, often use copyrighted materials without explicit permission from authors, leading to legal disputes over intellectual property rights. Samuelson (2023) analyzed how generative AI interacts with existing copyright laws, particularly in the U.S. and Europe, highlighting key challenges such as whether AI training on copyrighted content constitutes fair use, whether AI-generated outputs qualify as derivative works under copyright law, and who should bear legal responsibility for copyright violations—AI developers, users, or platforms. The study found that legal precedents remain unclear, with no consensus on whether using copyrighted material for AI training falls under fair use, creating significant regulatory uncertainty. Samuelson also emphasized the urgent need for updated copyright policies that define AI’s role in content creation, advocating for compensation models for creators whose works are used in AI training and stronger transparency requirements for AI dataset sources. Without clear legal frameworks, courts will struggle to regulate AI-generated works, potentially leading to widespread copyright disputes that challenge both intellectual property rights and AI innovation.

Beyond legal and academic concerns, AI also poses a significant environmental challenge due to the high energy consumption required for training and deploying large AI models. AI systems, particularly those used in healthcare and finance, contribute to substantial carbon emissions, raising ethical concerns about their sustainability. Richie (2022) emphasized that discussions on AI ethics often overlook its carbon footprint, arguing that environmental sustainability should be a core consideration in AI policy-making. The study proposed a framework for sustainable AI that integrates health, justice, and resource conservation principles into AI development. As AI adoption expands, researchers have called for energy-efficient AI models and greater transparency in AI-driven industries to mitigate environmental damage.

These ethical considerations are particularly relevant in discussions about AI’s role in shaping students’ career confidence and preparedness. While AI can possibly support skill development and career readiness, issues of academic dishonesty, copyright infringement, and environmental responsibility must be carefully managed to ensure AI serves as an equitable and sustainable tool.

## 3. Research Questions

As highlighted in the introduction, the release of ChatGPT marked a turning point in the adoption of artificial intelligence (AI) in education and career development. While ChatGPT and similar AI tools have demonstrated the potential to enhance learning, streamline workflows, and support career readiness, significant challenges remain. The introduction underscores pressing concerns, such as declining employment confidence among college students and the critical role of self-efficacy in career preparation and success. It also emphasizes how AI tools like ChatGPT can address these issues through personalized support and skill enhancement.

The literature review further detailed how ChatGPT has been shown to enhance cognitive skills, career-relevant knowledge, and self-efficacy. Additionally, studies highlight the importance of user experience as a mediator in determining the effectiveness of AI tools. However, despite these insights, gaps remain in understanding how ChatGPT influences employment confidence among diverse college student populations. Specifically, there is limited exploration of how these effects vary across academic levels (undergraduate, graduate, and doctoral students) and academic fields (social sciences, applied sciences, and arts and humanities). Furthermore, while user experience is acknowledged as critical, its specific mediating role in ChatGPT usage and employment confidence remains underexplored.

**Figure 1:**
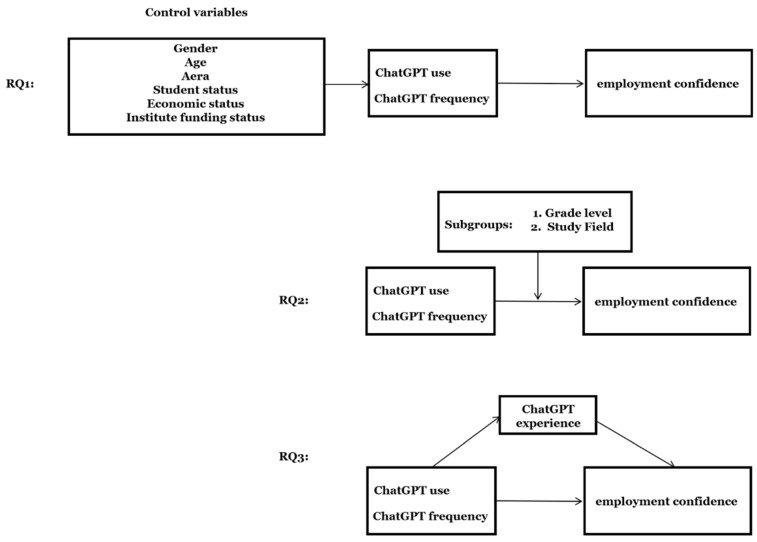
Research framework illustrating the three research questions.

To address these gaps, this study investigates the following research questions (see Figure 1):

RQ1: How does ChatGPT usage influence college students’ employment confidence?

RQ2: How does ChatGPT usage influence college students’ employment confidence across academic levels and fields?

RQ3: What roles do ChatGPT user experiences play in mediating its influence on college students’ employment confidence?

## 4. Methods

### 4.1. Data

This study utilized data from the Global ChatGPT Student Survey conducted by the Faculty of Public Administration, University of Ljubljana, aiming to understand how ChatGPT shapes higher education students’ experiences and learning outcomes by specifically analyzing how students with diverse cultural backgrounds view ChatGPT. In total, over 23,000 higher education students from 109 countries and territories—all at least 18 years old and currently enrolled in a higher education institution—were surveyed about their early experiences using ChatGPT in academic contexts. The survey employed an online questionnaire, administered via the 1KA (One Click Survey) platform, that comprised 42 primarily closed-ended questions (along with some open-ended ones) targeting higher education students’ initial experiences with ChatGPT. The questions were organized into themes exploring usage frequency, motivations for using ChatGPT, satisfaction and attitude toward ChatGPT as a learning tool, anticipated labor market implications, etc. Measured predominantly on 5-point Likert scales (e.g., “strongly disagree” to “strongly agree”), complemented by single-choice and open-ended items, this structure allowed for a detailed examination of how students across diverse cultural contexts perceive ChatGPT’s impact on their learning processes and future career prospects. The demographic characteristics of the participants are presented in Table 1.

### 4.2. Variables

#### 4.2.1. Dependent Variables

This study selects students’ confidence about getting a job as the dependent variable. In the Global ChatGPT Student Survey, students’ confidence about getting a job was measured with the question: “Do you feel confident about getting a job after you complete your studies?”. The specific items of the dependent variable are presented in Table 2.

#### 4.2.2. Independent Variables

This study introduces two independent variables, ChatGPT use and ChatGPT frequency, to describe participants’ utilization of ChatGPT. ChatGPT use is coded as 1 = Yes and 0 = No to indicate whether the participant has used ChatGPT. ChatGPT frequency is established to represent the frequency of ChatGPT use by the participants. The specific items of the independent variables are presented in Table 2.

#### 4.2.3. Control Variables

Research has confirmed that some demographic, economic, and social variables have an impact on individuals’ confidence in employment (Carlin et al., 2018; Zheng et al., 2022). Thus, this study employs gender (since only a few respondents selected “other” and “prefer not to disclose”, we are treating these responses as missing values), age, area, student status, economic status, and government funded as the control variables. The specific items of the control variables are presented in Table 2.

#### 4.2.4. Mediating Variable

This study employs students’ experience with ChatGPT as the mediating variable. In the Global ChatGPT Student Survey, students’ experience with ChatGPT was measured by the question: “What is your experience with ChatGPT?”. The specific items of the mediating variable are presented in Table 2.

### 4.3. Models

To explore the effect of ChatGPT on students’ employment confidence, this study constructs a regression model using STATA 17:

(1) $$\begin{array}{ll} Confidence= & \beta_{0}+\beta_{1}\cdot ChatGPTuse\times ChatGPTfrequency+\beta_{2}\cdot Gender+\beta_{3}\cdot Age\\ & +\beta_{4}\cdot Area+\beta_{5}\cdot SS+\beta_{6}\cdot ES+\beta_{7}\cdot GF+\varepsilon \end{array}$$

ChatGPTuse represents students’ utilization of ChatGPT; ChatGPTfrequency represents the frequency of ChatGPT use by students; Gender represents students’ gender; Age represents students’ age; Area represents the area students live in; SS represents student status; ES represents students’ economic status; GF represents whether students’ institution is funded by the government; β_0_ is the intercept; β_1_–β_7_ are the regression coefficients, and ε is the error term.

To address the endogeneity of the effect of ChatGPT use on students’ views on AI, this study employs Inverse Probability Weighting (IPW) to conduct a robustness test. IPW is a commonly used method in statistical analyses and causal inference to deal with selection bias or confounding variables in observational data. Its basic idea is to assign a weight to each observation so that the distribution of the treatment group and the control group on the confounding variable is balanced so that the treatment effect can be estimated more accurately (Cattaneo, 2010).

**Figure 2:**
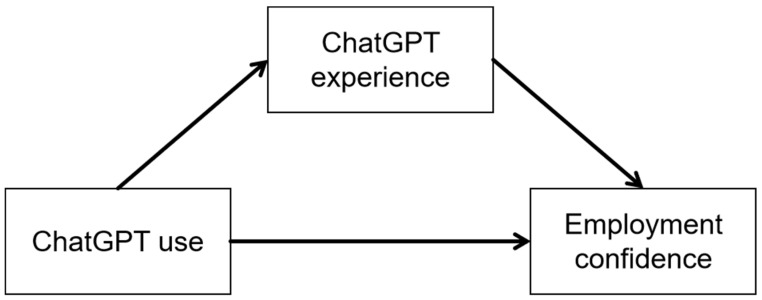
SEM pathway linking ChatGPT use to students’ employment confidence.

Subsequently, this study constructed a Structural Equation Modeling (SEM) framework (see Figure 2) to delve into the underlying mechanisms by which ChatGPT use influences students’ employment confidence utilizing STATA 18. To evaluate the model’s goodness of fit, the study employed the Comparative Fit Index (CFI), Tucker–Lewis Index (TLI), Root Mean Squared Error of Approximation (RMSEA), and Standardized Root Mean Squared Residual (SRMR) as metrics. An SEM model is considered to have an excellent fit when the CFI and TLI approach 1, while an RMSEA value below 0.08 and an SRMR value below 0.05 are indicative of an acceptable fit (Kline, 2005; Hair et al., 2006; Markus, 2012). The SEM model developed in this study yielded fit indices of CFI 0.999, TLI 0.979, RMSEA 0.017, and SRMR 0.003, indicating that the model provides an adequate representation of the intrinsic relationships embedded within the dataset.

## 5. Findings

### 5.1. Results of Regression Model

The results of the regression model (see Table 3) reveal that the interaction of ChatGPT use and ChatGPT frequency exerts a significant positive effect on students’ employment confidence (β = 0.050, *p* < 0.001), indicating that students who use ChatGPT have higher employment confidence than those who do not, and the more frequently they use ChatGPT, the higher their employment confidence. Additionally, age (β = 0.015, *p* < 0.001), area (β = 0.028, *p* = 0.045), student status (β = 0.136, *p* < 0.001), and economic status (β = 0.124, *p* < 0.001) have positive effects on students’ employment confidence, indicating that older students, those from urban areas, full-time students, and students with higher economic status have higher employment confidence. Moreover, gender (β = 0.091, *p* < 0.001) has a positive effect on students’ employment confidence, indicating that male students tend to have higher employment confidence than female students.

### 5.2. Results of Robustness Test

This study uses ChatGPT use (1 = Yes, 0 = No) as the grouping variable and the construct-treated group and control group through IPW to test the robustness of the effect of ChatGPT use on students’ employment confidence. The results of IPW (see Table 4) reveal that the use of ChatGPT has a significant positive average treatment effect on the treated (ATET) for students’ employment confidence (β = 0.044, *p* = 0.035), indicating that the findings of this study are robust.

### 5.3. Results of Heterogeneity Analysis

This study conducted a heterogeneity analysis of the effect of ChatGPT use on students’ employment confidence by categorizing students into different subgroups based on their study level and study field. The results of the heterogeneity analysis (see Table 5) suggest that the positive effect of ChatGPT use on undergraduate students’ employment confidence is statistically significant (β = 0.048, *p* < 0.001), whereas the effects on graduate students’ and doctoral students’ employment confidence are not statistically significant (*p* > 0.05). Additionally, the positive effect of ChatGPT use on students from social sciences (β = 0.048, *p* = 0.003) is significantly higher compared to students from applied sciences (β = 0.040, *p* = 0.009), while the effects on students from arts and humanities and natural and life sciences are not statistically significant (*p* > 0.05).

### 5.4. Results of SEM

**Figure 3:**
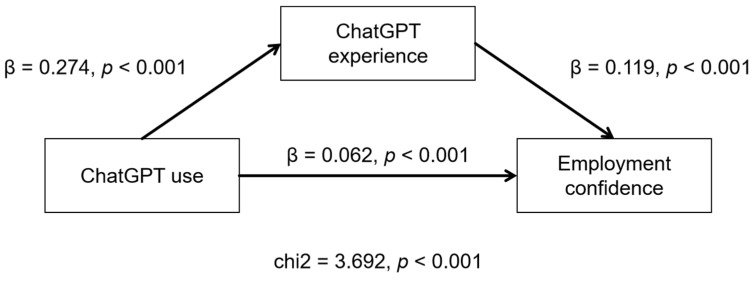
Results of SEM.

The results of SEM (see Figure 3) show that ChatGPT use has a significant positive direct effect on students’ employment confidence (β = 0.062, *p* < 0.001). The indirect effect of ChatGPT use on students’ employment confidence through ChatGPT experience is 0.033 (0.274 × 0.119), and this coefficient is statistically significant (*p* < 0.001). These findings suggest that ChatGPT experience partially mediates the relationship between ChatGPT use and students’ employment confidence.

## 6. Discussion

### 6.1. RQ1: The Role of ChatGPT in Enhancing Employment Confidence

The finding that ChatGPT usage significantly enhances students’ employment confidence aligns with broader discussions in the literature about the transformative role of AI tools in education and career readiness. ChatGPT, as a generative AI model, has demonstrated the ability to provide personalized, real-time support, which enhances students’ cognitive and career-relevant skills (Essel et al., 2024; Dahri et al., 2024). These capabilities, coupled with frequent usage, allow students to engage more deeply with learning materials and career preparation, fostering confidence in their employability. Additionally, this result resonates with studies emphasizing the importance of self-efficacy and job readiness in career success (Liu et al., 2014; Ristiani & Lusianingrum, 2022). As ChatGPT assists students in practical tasks such as resume building, interview preparation, and job search strategies, it contributes to building the skills necessary for navigating the job market effectively (Bhullar et al., 2024).

While ChatGPT offers substantial benefits, some studies also underscore critical drawbacks that warrant caution. Overreliance on ChatGPT may inadvertently diminish students’ intrinsic motivation and creativity, as observed in a reduced problem-solving initiative among frequent users (Muñoz et al., 2023; Toma & Yánez-Pérez, 2024). This aligns with concerns that AI tools could foster dependency, weakening the development of independent critical thinking—a skill vital for workplace adaptability. Furthermore, the tool’s uneven efficacy across disciplines risks reinforcing existing inequities; for instance, students in creativity-driven fields, like arts or empirical sciences, may perceive ChatGPT as irrelevant or even detrimental to their skill development (Trusz, 2020). Ethical dilemmas, such as academic integrity violations and job displacement anxieties, further complicate its adoption (Bin-Nashwan et al., 2023; Huseynov, 2023).

These findings highlight the pivotal role of access and usage frequency, suggesting that the equitable distribution of AI tools in education is essential to maximize their impact. At the same time, they advocate for balanced integration strategies that address both AI’s potential and its sociocognitive risks, ensuring students cultivate resilience alongside technological proficiency. To optimize ChatGPT’s role in career development, educational institutions must implement structured guidelines that promote its use as a supplement rather than a substitute for critical thinking and independent learning.

### 6.2. RQ2: Differences Across Academic Levels and Fields

The findings that ChatGPT has the most significant positive impact on undergraduate students and those in social sciences align with the existing literature on the role of technology in education. Undergraduates often rely heavily on technology for general academic and social activities (Kwon et al., 2013). This group may benefit more from ChatGPT’s accessibility and ease of use, which enhance their cognitive skills and career-relevant knowledge (Essel et al., 2024; Urban et al., 2024). These findings highlight how ChatGPT bridges gaps in academic support, particularly for younger students who might be less experienced with complex job market dynamics (Dahri et al., 2024).

However, the lack of statistically significant effects for graduate and doctoral students suggests that ChatGPT’s capabilities may not align as closely with the advanced, specialized needs of these groups. Graduate and doctoral students often prioritize targeted academic technology that supports in-depth research and professional development (Xu & Du, 2019). This gap underscores a limitation in how ChatGPT meets the nuanced demands of higher academic levels, emphasizing the need for more advanced features tailored to these users.

The findings also reveal distinct patterns across academic fields. The strongest positive impact on social sciences students may be attributed to advanced technologies’ ability to facilitate communication-intensive tasks, such as drafting, editing, and synthesizing information (Campos et al., 2014). In applied sciences, where the effects are moderate but significant, students likely benefit from these tools’ capabilities in problem-solving and analytical processes (Moses et al., 2014). However, the lack of significant effects for arts, humanities, and natural sciences students suggests a mismatch between these fields’ reliance on creativity or empirical research and the functionalities currently offered by such tools (Trusz, 2020).

These results also align with broader discussions on how advanced technologies impact career confidence. Their positive effects may stem from the ability to enhance self-efficacy in job-seeking tasks, a critical component of employment confidence (Liu et al., 2014). However, the absence of effects in certain groups highlights the importance of user trust, satisfaction, and field-specific applicability (Choudhury & Shamszare, 2023). For instance, students from underrepresented fields may not perceive technological tools as fully addressing their unique challenges, reflecting disparities in access and relevance of such innovations.

### 6.3. RQ3: The Mediating Role of User Experience in Enhancing Employment Confidence

The findings that users experience with ChatGPT partially mediate its impact on employment confidence, highlighting the critical role of interaction quality in leveraging AI tools for career readiness. This aligns with research emphasizing the importance of user trust and experience in determining the effectiveness of advanced technologies across various domains (Choudhury & Shamszare, 2023). A positive user experience facilitates greater self-efficacy, as students are more likely to feel confident in their ability to apply AI-generated insights in job-seeking tasks and career development.

This mediation effect also reflects broader discussions in the literature on how AI tools can enhance psychological factors tied to employability. Employment confidence is strongly influenced by self-efficacy and the perception of one’s skills and readiness to navigate the job market (Liu et al., 2014). ChatGPT’s ease of use and reliable outputs may contribute to these psychological drivers, enabling students to feel more equipped to tackle job-related challenges. Moreover, satisfaction derived from ChatGPT’s responsiveness and accuracy might translate into greater enthusiasm and optimism about employment prospects, as evidenced in research on adaptive learning environments and career tools (Bhullar et al., 2024; Essel et al., 2024).

### 6.4. Theoretical Explanations and Analytical Hypotheses for Future Research

Our findings indicate that ChatGPT usage is positively associated with students’ employment confidence, which can be understood through the lens of Social Cognitive Theory (Bandura, 1986). This theory suggests that individuals’ beliefs in their ability to succeed influence their behavior, and ChatGPT may enhance self-efficacy by providing immediate career advice, resume feedback, and interview preparation tools. Research has shown that AI-driven platforms, such as ChatGPT, can improve users’ self-efficacy by facilitating learning, reducing uncertainty, and increasing perceived competence in various domains (Patil & Pramod, 2024).

While our study primarily adopts a descriptive approach, it opens avenues for hypothesis-driven research. For instance, we hypothesize that the relationship between ChatGPT use and employment confidence may be mediated by perceived self-efficacy and moderated by students’ prior career knowledge. Studies have suggested that the effectiveness of AI tools in enhancing self-efficacy varies depending on users’ familiarity with career-related decision-making and digital literacy (Bui & Duong, 2024). Future research employing experimental or longitudinal designs could validate these relationships, providing deeper insights into the mechanisms underlying AI-assisted career support.

### 6.5. Context-Specific Insights and Generalizability

The findings of this study reflect both contextual nuances and broader trends relevant to AI-driven career development. While ChatGPT’s benefits are widely recognized, their effects may differ based on regional labor markets and AI adoption levels. For instance, in developing economies, where job market instability is a key concern, ChatGPT’s potential to enhance employment confidence may be constrained by limited access to digital career tools (James, 2021). However, studies show that AI can play a transformative role by improving self-efficacy and job readiness, particularly for students who lack traditional career support resources (Patil & Pramod, 2024). This suggests that while AI’s effectiveness varies across contexts, its potential to enhance career development is broadly applicable. To foster international relevance, future research should explore how AI’s role in employment confidence differs across diverse educational and economic landscapes. Comparative studies on AI adoption in career services across regions with varying labor market dynamics would provide deeper insights into its global applicability and limitations.

## 7. Conclusions

Addressing RQ1: How does ChatGPT usage influence college students’ employment confidence?

This study advances empirical evidence by establishing that ChatGPT usage directly enhances college students’ employment confidence, particularly through regular and sustained engagement. The causal relationship, rigorously validated via Inverse Probability Weighting (IPW), demonstrates a significant positive average treatment effect, confirming that students who actively use ChatGPT exhibit greater confidence in securing employment than non-users. This finding extends the prior literature on AI in education (e.g., Hakiki et al., 2023) by isolating ChatGPT’s unique role in career preparedness, moving beyond generic discussions of AI tools to quantify its specific impact on confidence building. Importantly, this study identifies frequency of use as a critical moderator, suggesting that habitual interaction amplifies benefits—a nuance absent in earlier research.

Addressing RQ2: How does ChatGPT usage influence employment confidence across academic levels and fields?

By conducting a heterogeneity analysis, this work reveals that ChatGPT’s impact is not uniform but varies significantly across academic levels and disciplines. Undergraduate students and those in social sciences derive disproportionately higher confidence gains compared to graduate students or those in technical fields. This finding challenges the assumption of universal AI tool efficacy and highlights the need for context-specific integration strategies. For instance, tailored ChatGPT applications in social sciences (e.g., simulating job interviews or resume-building exercises) may explain the heightened benefits, whereas technical fields might require more specialized AI adaptations. These insights refine frameworks for AI adoption in education, emphasizing disciplinary and academic-level disparities—a dimension underexplored in existing studies (Chen et al., 2023).

Addressing RQ3: What roles do ChatGPT user experiences play in mediating its influence on employment confidence?

The Structural Equation Modeling (SEM) results provide novel insights into the mediating mechanism, showing that positive ChatGPT experiences (e.g., perceived usefulness, ease of use, and response quality) partially mediate the relationship between usage and confidence. This aligns with and extends the user experience theories of Ngo et al. (2024), demonstrating that confidence gains depend not only on tool adoption but also on subjective satisfaction. For example, students who found ChatGPT intuitive and reliable reported stronger confidence improvements, underscoring the importance of optimizing AI interfaces and outputs. This mediation analysis bridges a critical gap in the literature, shifting the focus from mere usage metrics to qualitative user experiences as drivers of AI’s educational value.

## 8. Implication

The findings of this study carry several important implications for educational institutions, policymakers, and students. Firstly, the significant positive effect of ChatGPT use on students’ employment confidence underscores the potential of AI tools to enhance educational experiences and prepare students for the job market. Research indicates that ChatGPT enhances learning outcomes by providing personalized feedback, simplifying complex topics, and increasing accessibility to education for underserved populations (Altarawneh, 2023). Additionally, ChatGPT contributes to student satisfaction and engagement, as its ease of use and perceived usefulness encourage continued utilization and support knowledge acquisition (Ngo et al., 2024).

Secondly, this study’s indication that the benefits of ChatGPT use are more pronounced for undergraduate students and those in the social sciences suggests that AI interventions may need to be tailored to different educational levels and disciplines. This could lead to more targeted AI education programs that address the specific needs of various student populations. AI-based personalized e-learning systems can deliver adaptive and adaptable content tailored to individual learners’ comprehension and preferences, enhancing learning outcomes and addressing diverse educational needs (Murtaza et al., 2022).

Furthermore, the findings highlight the importance of addressing potential disparities in AI literacy, as students from urban areas and those with higher economic status appear to benefit more from ChatGPT use. The development and application of AI systems often reveal disparities, especially when access to technology and knowledge is unequally distributed, making it crucial to bridge these gaps for more equitable AI benefits (Schiff et al., 2021). This also underscores the need for equitable access to AI tools and resources in education.

Lastly, the partial mediation of ChatGPT experience suggests that hands-on interaction with AI can be a valuable component in building employment confidence. Generative AI tools, like ChatGPT, offer tailored and immersive learning experiences that simulate professional challenges, fostering skills and confidence necessary for career success (Mittal et al., 2024). Therefore, educational institutions should focus on providing opportunities for students to engage meaningfully with AI to develop the necessary skills and experiences for their future careers.

## 9. Limitations and Future Directions

This study has several limitations that should be considered. Firstly, the data were collected through self-reported surveys, which may be subject to biases, such as social desirability and recall bias. Secondly, the sample may not be fully representative of the global student population, as it was drawn from a specific set of universities. Thirdly, while the study controls for various demographic factors, it does not account for all potential confounding variables that could influence employment confidence, such as cultural background or personal networks. Lastly, the current study focuses on the relationship between ChatGPT use and employment confidence without examining the quality of the interactions with ChatGPT or the specific ways in which students are using the tool.

In terms of future directions, it would be valuable to investigate the long-term impacts of AI tools, like ChatGPT, on students’ career trajectories and job performance. Additionally, research could explore the development of tailored AI educational programs that cater to the diverse needs of students from different disciplines and backgrounds. Furthermore, the potential ethical implications of AI use in education should be examined to ensure that these tools are used responsibly and equitably.

## Figures and Tables

**Table 1:** Demographic table.

Demographic Characteristic	Declaration		N	Percentage
Gender	Male		9279	40.59%
	Female		13,247	57.95%
	Other		103	0.45%
	Prefer not to disclose		230	1.01%
Area	Urban		11,326	64.33%
	Suburban		3481	19.77%
	Rural		2799	15.90%
Student status	Full-time		19,266	85.27%
	Part-time		3327	14.73%
Study level	Undergraduate		18,784	83.31%
	Postgraduate		2856	12.67%
	Doctoral		906	4.02%
Study field	Arts and humanities		2696	12.01%
	Social sciences		9290	41.37%
	Applied sciences		7764	34.58%
	Natural and life sciences		2705	12.05%
Economic status	Significantly below-average		1172	6.65%
	Below-average		3480	19.74%
	Average		9830	55.75%
	Above-average		2728	15.47%
	Significantly above-average		423	2.40%
Variables	Mean	S.D.	Max	Min
Age	23.13	6.82	100	18

**Table 2:** List of survey items and corresponding constructs.

Dependent Variable	Question/Items	Options
Confidence about getting a job	Do you feel confident about getting a job after you complete your studies?	1 = Not at all confident 2 = Slightly confident 3 = Moderately confident 4 = Very confident 5 = Extremely confident
Independent variable	Question/Items	Options
ChatGPT use	Have you ever used ChatGPT?	1 = Yes 0 = No
ChatGPT frequency	To what extent do you use ChatGPT in general?	1 = Rarely 2 = Occasionally 3 = Moderately 4 = Considerably 5 = Extensively
Control variable	Question/Items	Options
Gender	What is your gender?	1 = Male 0 = Female
Age	How old are you (in years)?	
Area	Which of the characteristics below describes the area you live in?	1 = Rural 2 = Suburban 3 = Urban
Student status	What is your student status?	1 = Full-time 0 = Part-time
Economic status	What is your economic status?	1 = Rarely 2 = Occasionally 3 = Moderately 4 = Considerably 5 = Extensively
Government funded	Is your institution publicly/government funded?	1 = Yes 0 = No
Mediating variable	Question/Items	Options
ChatGPT experience	What is your experience with ChatGPT?	1 = Very bad 2 = Bad 3 = Neutral 4 = Good 5 = Very good

**Table 3:** Results of regression analysis.

Independent Variable	β	S.E.	*t*	*p*	95% Conf. Interval	
ChatGPTuse × ChatGPTfrequency	0.050	0.010	5.15	<0.001	0.031	0.069
Gender	0.091	0.021	4.25	<0.001	0.049	0.132
Age	0.015	0.002	8.25	<0.001	0.011	0.019
Area	0.028	0.014	2.01	0.045	0.001	0.056
Student status	0.136	0.033	4.08	<0.001	0.071	0.201
Economic status	0.124	0.013	9.72	<0.001	0.099	0.149
Government fund	−0.023	0.026	−0.87	0.386	−0.073	0.028
Constant	2.290	0.082	27.86	<0.001	2.129	2.451
N = 9384 R^2^ = 0.0249						

**Table 4:** Results of IPW.

	β	S.D.	*z*	*p*	95% Conf. Interval	
ATET chatgptuse: yes vs. no	0.044	0.021	2.11	0.035	0.003	0.084
Pomean chatgptuse: no	3.292	0.018	184.38	<0.001	3.257	3.327
N = 13,635						

**Table 5:** Results of heterogeneity analysis.

Subgroup	Independent Variable	β	S.E.	*t*	*p*	95% Conf. Interval	
Undergraduate (N = 7838, R^2^ = 0.0183)	ChatGPTuse × ChatGPTfrequency	0.048	0.011	4.52	<0.001	0.027	0.069
	Gender	0.083	0.023	3.58	<0.001	0.037	0.128
	Age	0.013	0.003	4.87	<0.001	0.008	0.018
	Area	0.024	0.015	1.61	0.108	−0.005	0.054
	Student status	0.206	0.038	5.43	<0.001	0.131	0.280
	Economic status	0.102	0.014	7.32	<0.001	0.075	0.129
	Government fund	−0.023	0.028	−0.84	0.403	−0.078	0.032
	Constant	2.343	0.098	24.03	<0.001	2.152	2.534
Graduate (N = 1095, R^2^ = 0.0426)	ChatGPTuse × ChatGPTfrequency	0.038	0.029	1.31	0.189	−0.019	0.094
	Gender	0.202	0.065	3.12	0.002	0.075	0.329
	Age	−0.000	0.004	−0.08	0.933	−0.009	0.008
	Area	0.053	0.043	1.21	0.226	−0.033	0.138
	Student status	−0.126	0.085	−1.49	0.136	−0.293	0.040
	Economic status	0.196	0.038	5.15	<0.001	0.121	0.270
	Government fund	−0.035	0.082	−0.43	0.670	−0.197	0.127
	Constant	2.753	0.238	11.57	<0.001	2.286	3.220
Doctoral (N = 414, R^2^ = 0.0625)	ChatGPTuse × ChatGPTfrequency	0.077	0.048	1.60	0.111	−0.018	0.171
	Gender	0.119	0.105	1.13	0.260	−0.088	0.325
	Age	0.004	0.006	0.68	0.495	−0.008	0.016
	Area	−0.026	0.081	−0.33	0.743	−0.185	0.132
	Student status	−0.052	0.138	−0.38	0.706	−0.323	0.219
	Economic status	0.277	0.068	4.05	<0.001	0.142	0.411
	Government fund	−0.215	0.142	−1.52	0.130	−0.493	0.064
	Constant	2.712	0.423	6.41	<0.001	1.880	3.544
Arts and humanities (N = 1132, R^2^ = 0.0188)	ChatGPTuse × ChatGPTfrequency	0.037	0.030	1.22	0.222	−0.022	0.095
	Gender	−0.041	0.068	−0.60	0.546	−0.174	0.092
	Age	0.021	0.005	4.11	<0.001	0.011	0.032
	Area	0.027	0.040	0.66	0.508	−0.052	0.105
	Student status	0.070	0.090	0.78	0.438	−0.107	0.246
	Economic status	−0.023	0.037	−0.63	0.531	−0.095	0.049
	Government fund	−0.124	0.088	−1.41	0.159	−0.296	0.049
	Constant	2.522	0.236	10.70	<0.001	2.060	2.984
Social sciences (N = 3761, R^2^ = 0.0282)	ChatGPTuse × ChatGPTfrequency	0.048	0.016	2.99	0.003	0.016	0.079
	Gender	0.023	0.035	0.67	0.500	−0.044	0.091
	Age	0.017	0.003	6.53	<0.001	0.012	0.022
	Area	0.028	0.021	1.31	0.189	−0.014	0.069
	Student status	0.174	0.050	3.51	<0.001	0.077	0.272
	Economic status	0.136	0.020	6.75	<0.001	0.097	0.176
	Government fund	−0.023	0.040	−0.57	0.566	−0.100	0.055
	Constant	2.164	0.123	17.52	<0.001	1.922	2.406
Applied sciences (N = 3435, R^2^ = 0.0271)	ChatGPTuse × ChatGPTfrequency	0.040	0.015	2.62	0.009	0.010	0.070
	Gender	0.085	0.034	2.47	0.013	0.018	0.152
	Age	0.015	0.003	4.24	<0.001	0.008	0.022
	Area	0.012	0.025	0.47	0.642	−0.037	0.060
	Student status	0.048	0.061	0.79	0.428	−0.071	0.167
	Economic status	0.160	0.021	7.61	<0.001	0.119	0.201
	Government fund	0.003	0.041	0.07	0.948	−0.078	0.083
	Constant	2.439	0.148	16.46	<0.001	2.148	2.729
Natural and life sciences (N = 1007 R^2^ = 0.0113)	ChatGPTuse × ChatGPTfrequency	0.027	0.030	0.89	0.375	−0.032	0.086
	Gender	0.100	0.066	1.52	0.130	−0.030	0.230
	Age	0.002	0.006	0.32	0.747	−0.009	0.013
	Area	−0.002	0.042	−0.05	0.958	−0.084	0.080
	Student status	0.122	0.102	1.20	0.231	−0.078	0.322
	Economic status	0.102	0.040	2.56	0.011	0.024	0.180
	Government fund	0.001	0.093	0.01	0.991	−0.181	0.183
	Constant	2.851	0.253	11.25	<0.001	2.353	3.348

## Data Availability

The data presented in this study are available on http://doi.org/10.17632/ymg9nsn6kn.1.
